# Exome Sequencing in a Family with Luminal-Type Breast Cancer Underpinned by Variation in the Methylation Pathway

**DOI:** 10.3390/ijms18020467

**Published:** 2017-02-22

**Authors:** Nicole van der Merwe, Armand V. Peeters, Fredrieka M. Pienaar, Juanita Bezuidenhout, Susan J. van Rensburg, Maritha J. Kotze

**Affiliations:** 1 Division of Anatomical Pathology, Department of Pathology, Faculty of Medicine and Health Sciences, Stellenbosch University, Tygerberg 7500, South Africa; vmrnic015@myuct.ac.za (N.v.d.M.); avpeeters@sun.ac.za (A.V.P.); jbez@sun.ac.za (J.B.); 2 GVI Oncology, Mediclinic Panorama, Cape Town 8000, South Africa; rika.pienaar@cancercare.co.za; 3 Division of Chemical Pathology, Department of Pathology, Faculty of Medicine and Health Sciences, Stellenbosch University, Tygerberg 7500, South Africa; sjvr@sun.ac.za; 4 National Health Laboratory Service, Tygerberg Hospital, Tygerberg 7500, South Africa

**Keywords:** breast cancer, exome sequencing, methylation pathway, pathology, pharmacogenomics

## Abstract

Panel-based next generation sequencing (NGS) is currently preferred over whole exome sequencing (WES) for diagnosis of familial breast cancer, due to interpretation challenges caused by variants of uncertain clinical significance (VUS). There is also no consensus on the selection criteria for WES. In this study, a pathology-supported genetic testing (PSGT) approach was used to select two *BRCA1/2* mutation-negative breast cancer patients from the same family for WES. Homozygosity for the *MTHFR* 677 C>T mutation detected during this PSGT pre-screen step was considered insufficient to cause bilateral breast cancer in the index case and her daughter diagnosed with early-onset breast cancer (<30 years). Extended genetic testing using WES identified the *RAD50* R385C missense mutation in both cases. This rare variant with a minor allele frequency (MAF) of <0.001 was classified as a VUS after exclusion in an affected cousin and extended genotyping in 164 unrelated breast cancer patients and 160 controls. Detection of functional polymorphisms (MAF > 5%) in the folate pathway in all three affected family members is consistent with inheritance of the luminal-type breast cancer in the family. PSGT assisted with the decision to pursue extended genetic testing and facilitated clinical interpretation of WES aimed at reduction of recurrence risk.

## 1. Introduction

Oncogenomics is a rapidly evolving integrative discipline that enables a novel approach to the molecular characterization of breast cancer pathology. Understanding the relationship between germline and tumour genetics is important in patients where early-onset familial breast cancer or differences in drug response and survival outcome cannot be explained by mutations in the major tumour suppressor genes, *BRCA1* and *BRCA2* [1,2,3]. In these circumstances, genome-scale microarray analysis and/or next generation sequencing (NGS) provide viable options for comprehensive testing in a well-defined target population. Application of these advanced molecular technologies in the diverse South African population evolved into a pathology-supported genetic testing (PSGT) service used to facilitate the move from single- to multi-gene testing and NGS [4]. Utilization of PSGT may extrapolate the capacity of NGS from genotype to phenotype.

NGS includes targeted sequencing of preselected gene panels as well as whole genome/exome sequencing, RNA sequencing, and bisulfite sequencing for assessment of DNA methylation patterns [5,6]. Although targeted sequencing panels are currently the method of choice in breast cancer diagnostics, we favour the use of whole exome sequencing (WES) preceded by PSGT to facilitate patient selection and clinical interpretation [7]. WES is not limited by the number of genes that can be evaluated for risk of familial breast cancer and tailored therapeutic intervention in a single test. However, the large amount of information that is generated throughout the coding region of the genome may outpace our ability to adequately interpret variant pathogenicity for clinical application. Comprehensive studies on the pathology of hereditary breast cancer is needed to characterize variants of uncertain clinical significance (VUS) increasingly uncovered by WES [8,9], which may use lower thresholds for breast cancer mutation screening compared to targeted *BRCA1/2* mutation screening. There is currently no consensus on eligibility criteria for WES in *BRCA1/2* mutation-negative familial breast cancer patients.

Grant et al. [10] demonstrated the value of a pre-screen algorithm using PSGT to reduce chemotherapy overtreatment in South African breast cancer patients eligible for microarray analysis using the 70-gene MammaPrint test. A similar approach was validated in the Microarray in Node-negative Disease may Avoid ChemoTherapy (MINDACT) trial, which provided level 1A evidence for the clinical utility of MammaPrint. Early-stage breast cancer patients classified as clinical high-risk based on tumour size, grade, nodal status, estrogen receptor (ER) and human epidermal growth factor receptor 2 (HER2) status, are eligible for microarray analysis [11]. The fact that approximately 50% of early-stage breast cancer patients at high risk of recurrence could safely forego chemotherapy based on a low-risk MammaPrint profile, confirmed the important role of genomics to stratify breast cancer patients into different treatment groups. The finding that susceptibility to a particular molecular subtype of breast cancer is associated with familial aggregation [8,12] furthermore emphasised the importance of MammaPrint used in conjunction with the 80-gene Blueprint microarray to subdivide tumours into the Luminal A, Luminal B, HER2-enriched and basal-types [13,14].

Germline genotyping interpreted in the context of tumour subtype may help to determine whether breast cancer is caused by genetic or environmental factors, or a combination of both, in order to direct treatment accordingly. Naushad et al. [15] found that the Luminal A subtype is associated with lack of family history of breast cancer, late age of onset and high body mass index (BMI), in contrast to the basal-type known to be strongly associated with high-penetrance *BRCA1* familial breast cancer [8]. In line with tumour pathology, genes involved in breast cancer can broadly be categorized into those with high, moderate, and low penetrance of clinical manifestation. High-penetrance alleles typically confer lifetime risks of breast cancer above 50%, while moderate penetrance alleles confer risks greater than 20% and low-penetrance alleles increases of 10%–20% lifetime risk [16]. Risk above 50% is sufficient to recommend prophylactic surgery or magnetic resonance imaging screening in a familial context. Risk reduction strategies based on low-moderate penetrance genes are focused more on individual breast cancer survivors, where identification of biomarkers representing critical biological processes may be targeted at the gene–environment level to reduce the incidence of breast cancer recurrence.

It is estimated that known breast cancer susceptibility loci including *BRCA1* and *BRCA2* account for approximately 20% of familial risk, with the remaining variation caused by mutations with low-moderate penetrance that may be inherited in a subtype specific manner [17,18]. Failure to show significant survival differences between patients with familial and sporadic breast cancer [19] may partly be explained by polymorphic variation in drug metabolizing enzymes such as cytochrome P450 (CYP) [20], or modifier genes dependent on nutrient co-factors. Folate and other B-vitamins are important determinants of genome stability, yet might also contribute to the mechanism of action of anti-folates and fluoropyrimidines used in cancer treatment [21,22]. Xu et al. [22] identified one-carbon metabolism as a key pathway to be targeted for improved breast cancer survival.

Germline variation in folate pathway genes is associated with aberrant patterns of tumour pathology in cancer patients [23], with dysfunction of one-carbon metabolism found to be one of the three most important disease mechanisms in the pathogenesis of cancer [24]. Growing evidence of the complex interaction between methyl-related nutrients, alcohol and genetic variation in the methylene tetrahydrofolate reductase (*MTHFR*) gene support individualised dietary recommendations to reduce cancer risk in the general population [22,25]. These include substitution of low folate foods with folate-dense fruits and vegetables in countries where there is no mandatory folic acid fortification. Folate provides protection against carcinogenesis, especially in *MTHFR* mutation-positive cases, yet high doses of the synthetic form (folic acid) are suspected to promote tumour growth when administered after diagnosis of cancer. It is therefore reassuring that recent evidence obtained in postmenopausal patients with increased folate intake through diet and/or supplementation showed significantly higher circulating folate concentrations associated with improved survival after breast cancer diagnosis [26]. Since the negative effects of low intakes of the methyl-related nutrients with high intakes of alcohol are additive, Bailey [25] suggested that alcohol intake should be restricted to less than 15 g per day, if consumed.

The low-penetrance *MTHFR* 677 C>T mutation found to be associated with hormone receptor positive breast cancer [15], has previously been linked with increased BMI in South African patients diagnosed with major depressive disorder [27]. A low folate score, as reflected by the nutrition questionnaire used by Delport et al. [27], correlated with increased BMI and risk of depression in South African patients with the *MTHFR* 677 C>T mutation. Depression is a major comorbidity in breast cancer survivors and impacts on both the quality of life and treatment decision making. In this context, Van der Merwe et al. [28] recommended *CYP2D6* genotyping in breast cancer patients who (1) receive tamoxifen treatment; and (2) are at increased recurrence risk due to a family history of breast cancer/*BRCA*-mutation positivity; or (3) are required to take potential competing antidepressants that may inhibit *CYP2D6* enzyme function. *CYP2D6* genotyping combined with the unique spectrum of *BRCA1/2* founder mutations identified in the South African population [29,30], forms part of the pathology-supported pharmacogenetics test developed by van der Merwe [31] for improved clinical management of patients with breast cancer and associated comorbidities. Combining clinico-pathological features with pharmacogenetics in this chronic disease screening test is supported by the findings of Phipps et al. [32,33], who demonstrated subtype association with a family history of cancer and high BMI.

In this study, two *BRCA1/2* mutation-negative breast cancer patients were selected from the same family for WES, followed by extended mutation screening in 164 unrelated cases stratified according to ER status. Lifestyle risk factors such as BMI were assessed in study participants due to the link with cardiovascular disease (CVD), predicted to equal or exceed recurrence risk of breast cancer in ER-positive postmenopausal women [34]. This approach can be extended into a PSGT pre-screen algorithm for WES, to help distinguish between familial breast cancer eligible for NGS and patients with lifestyle-related breast cancer who may reduce their recurrence risk by maintaining a healthy body weight, smoking cessation and alcohol restriction [35]. These lifestyle factors may act in combination with low-penetrance mutations underlying the folate-vitamin B12-methylation pathway evaluated as part of a CVD multi-gene assay applied in this study before commencing WES [4]. Testing for *MTHFR* 677 C>T as an invariant marker of increased risk for many chronic diseases associated with dysfunctional methylation may add value beyond biochemical analysis of homocysteine and folate levels, which are influenced by various environmental factors. For this reason, *MTHFR* is not only a feasible anticancer drug target, but may also be considered an important factor in prevention of cumulative disease risk across clinical entities.

## 2. Results

### 2.1. Pathology-Supported Genetic Testing (PSGT)

**Figure 1:**
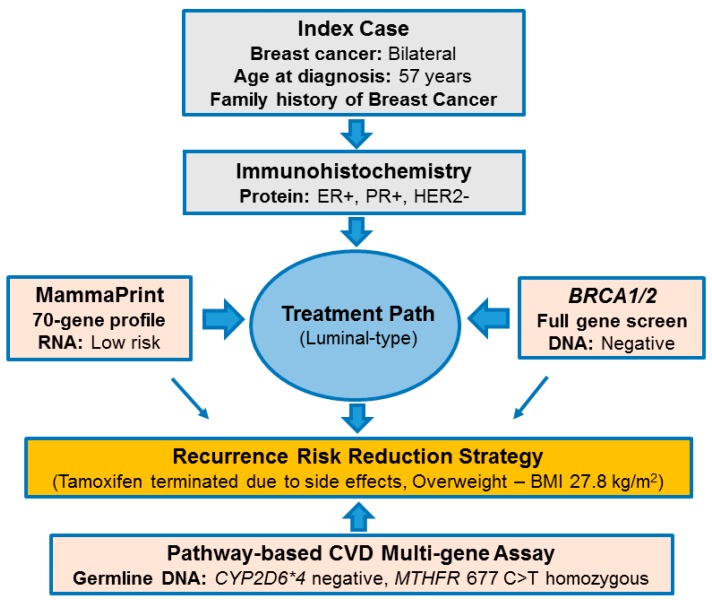
Outline of the pathology-supported genetic testing (PSGT) strategy used to select the index case with bilateral *BRCA1/2* mutation-negative breast cancer for whole exome sequencing (WES). Estrogen receptor (ER), progesterone receptor (PR) and human epidermal growth factor receptor-2 (HER2) status was determined using immunohistochemistry (IHC). These features, including tumour size, grade and nodal status were evaluated to determine eligibility for MammaPrint. Based on the low-risk recurrence score obtained with this microarray test, tamoxifen treatment was administered without addition of chemotherapy. Termination of tamoxifen after one year due to adverse drug response led to pharmacogenetic *CYP2D6* genotyping, which was performed as part of a chronic disease screen incorporating the cardiovascular disease (CVD) multi-gene assay. Detection of the methylenetetrahydrofolate reductase (*MTHFR*) 677 C>T mutation identified dysfunction of the folate pathway as a potential target for recurrence risk reduction in this overweight patient. Evaluation of family history, clinical characteristics, and laboratory test results at the protein (IHC), RNA (MammaPrint) and DNA (*BRCA1/2*, CVD multi-gene assay) levels supported the decision to perform extended genetic testing using WES. BMI: body mass index; CYP: cytochrome P450.

Figure 1 illustrates the PSGT approach used to select the index case for WES. Despite bilateral and early-onset breast cancer in the family, this patient tested negative for high-penetrance mutations in the *BRCA1* and *BRCA2* genes. She was initially referred for the MammaPrint test and could safely forego chemotherapy based on a low genomic risk for breast cancer recurrence. Microarray analysis was preceded by immunohistochemistry (IHC) testing indicating ER-positive (ER+), progesterone receptor-positive (PR+) and HER2-negative (HER2−) status. The index case remained disease-free since 2008 despite termination of tamoxifen after one year due to severe side effects, including. Use of the CVD multi-gene test excluded the most prevalent *CYP2D6* null-allele in Caucasians (CYP2D6*4), while homozygosity of *MTHFR* 677 C>T was detected in both the index case and her daughter with the luminal-type breast cancer. It was uncertain whether this functional single nucleotide polymorphism (SNP) expressed in a high-risk environment, as reflected by high BMI (>27 kg/m^2^) and inadequate folate intake (score <14) in both cases, was sufficient for development of breast cancer in this family.

### 2.2. Comprehensive Cancer Panel Screen Using Whole Exome Sequencing

**Figure 2:**
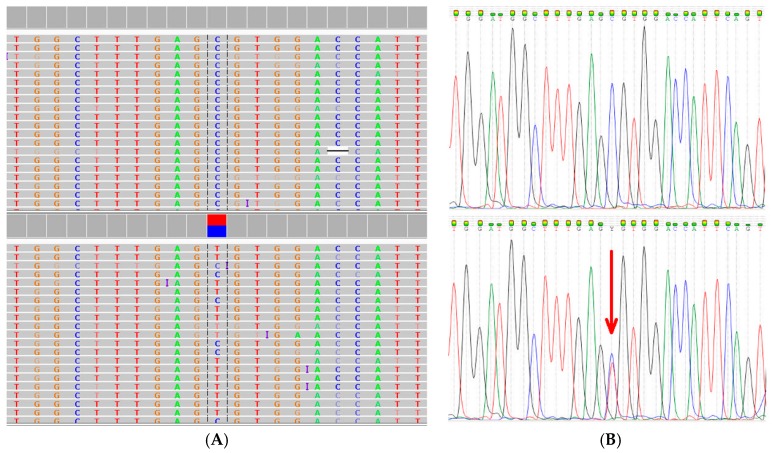
Detection of the *RAD50* missense mutation R385C (rs139372231, Refseq mRNA accession NM_005732.3) using whole exome sequencing (**A**); and visualized using the Integrative Genome Viewer software tool [36]. The C to T base change at nucleotide position 1153 was confirmed by Sanger sequencing (**B**) as indicated by the arrow.

**Figure 3:**
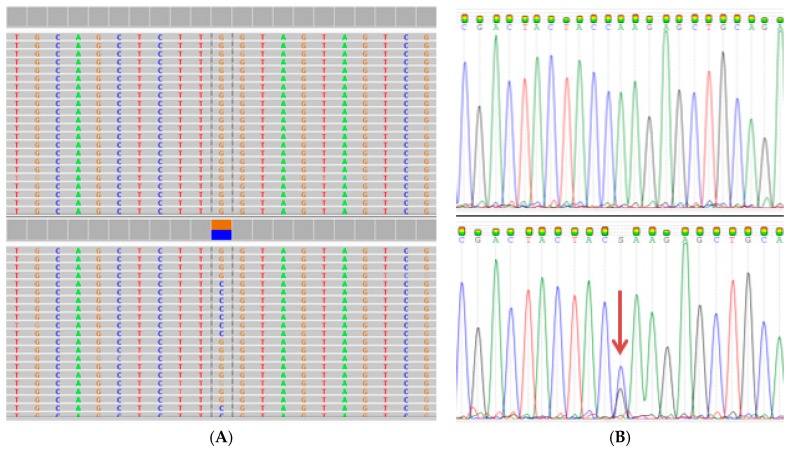
Detection of the *MUC1* missense mutation Q85E (rs773704188, Refseq mRNA accession NM_002456.5) using WES (**A**); and visualized using the Integrative Genome Viewer software tool [36]. The C to G base change at nucleotide position 325 was confirmed by Sanger sequencing (**B**) as indicated by the arrow.

WES was used to search for potentially causative gene variants in 409 known cancer-related genes included in the Ion AmpliSeq™ Comprehensive Cancer Panel (ThermoFisher, Waltham, MA, USA). Rare variants in the double strand break repair protein (*RAD50* R385C, Refseq mRNA accession NM_005732.3) and mucin 1 (*MUC1* Q85E, Refseq mRNA accession NM_002456.5) genes were identified as potential causative mutations. These missense mutations were confirmed using Sanger sequencing (Figure 2 and Figure 3).

The *RAD50* R385C mutation was detected in both the index case and her affected daughter, while the *MUC1* Q85E mutation was only identified in the daughter. Both mutations were absent in a cousin of the index case diagnosed with breast cancer at the age of 42 years. Unlike *RAD50*, *MUC1* has not been classified as a driver gene in breast cancer and was therefore not included in further analysis. Bioinformatic analysis of *RAD50* R385C with a minor allele frequency (MAF) of <0.001 is shown in Table 1. Three of the seven software tools predicted a disease-causing damaging effect and one a moderate impact.

### 2.3. Extended Mutation Analysis

Extended mutation screening for *RAD50* R385C using allele-specific real-time polymerase chain reaction (PCR) methodology excluded this rare variant in an additional 163 unrelated breast cancer patients stratified according to ER status (49 ER-negative, 114 ER-positive). This missense mutation was however detected in one (aged 36 years without a family history of cancer) of the 160 control individuals screened, but absent in the cousin of the index case diagnosed with lobular invasive carcinoma at the age of 42 years.

**Figure 4:**
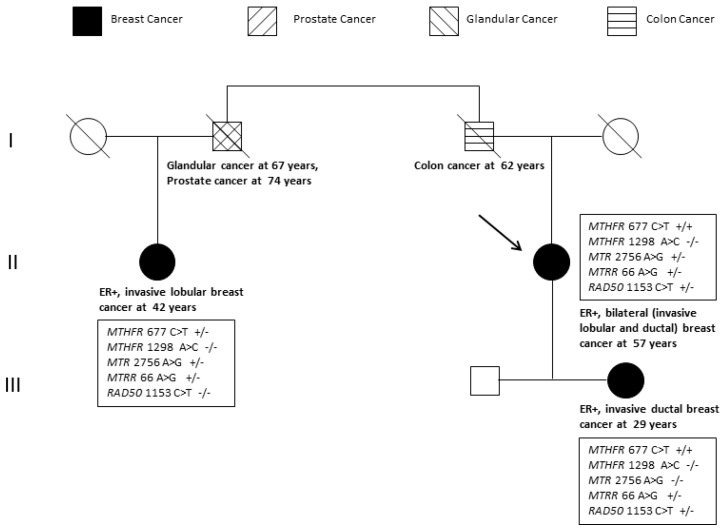
Pedigree of the index patient (arrow) subjected to whole exome sequencing, depicting the clinical (age of onset), pathology (tumour characteristics) and genetic heterogeneity in the family. Homozygosity for a minor allele is indicated by (+/+) and heterozygosity by (+/−). ER+: estrogen receptor-positive.

Failure to identify high impact pathogenic mutations in all affected family members prompted extended analysis for functional polymorphisms (minor allele frequency > 1%) in the folate pathway genes, methionine synthase (*MTR*) and methionine synthase reductase (*MTRR).* These genes are included in the Ion AmpliSeq™ Comprehensive Cancer Panel used to identify the *RAD50* and *MUC1* rare variants. Two functional SNPs were detected in the *MTR* (2756 A>G, rs1805087) and *MTRR* (66 A>G, rs1801394) genes, in addition to the low-penetrance *MTHFR* 677 C>T (rs1801133) mutation previously identified using the testing algorithm illustrated in Figure 1. *MTHFR* 1298 A>C (rs1801131) known to be in linkage disequilibrium with *MTHFR* 677 C>T was not detected. Extended analysis of the *CYP2D6* gene in the index case due to premature termination of tamoxifen, confirmed a fully functional enzyme based on the gene region covered by WES. Figure 4 illustrates the clinical, histopathological and genetic heterogeneity in the index patient, her daughter and cousin.

## 3. Discussion

After excluding high-penetrance mutations in the coding regions of the *BRCA1* and *BRCA2* genes, as well as screening for relatively common low-penetrance SNPs in key disease pathways underlying genomic instability (*MTHFR* 677 C>T and 1298 A>C) and drug metabolism (CYP2D6*4), WES was performed in the index patient and her daughter. An important aim was to determine whether homozygosity for the *MTHFR* 677 C>T mutation may be sufficient to cause ER-positive breast cancer in a high-risk environment as reflected by high BMI in both patients. WES identified a rare missense mutation R385C in the DNA mismatch repair gene *RAD50*, in both the mother and daughter. This rare variant was excluded in a cousin diagnosed with invasive lobular carcinoma at the age of 42 years, as well as a further 163 unrelated breast cancer patients selected from the genomics database. The index case was diagnosed with bilateral breast cancer, invasive ductal and lobular carcinoma, while the daughter was diagnosed with invasive ductal carcinoma. It is therefore possible that *RAD50* R385C may only be associated with invasive ductal carcinoma. However, detection of *RAD50* R385C in one of 160 controls screened and conflicting results obtained with use of several bioinformatics tools to predict functionality, classifies this missense mutation as a VUS.

Aloraifi et al. [37] identified *RAD50* mutations in approximately 3% of genetically uncharacterised breast cancer in a cohort of non-*BRCA* cases analysed. These authors reported potentially pathogenic variants that may explain 35% of the missing heritability. Bodmer and Tomlinson [38] suggested that whether or not rare variants end up filling the heritability gap, it is imperative to search for rare variants alongside common variants. *RAD50* has been documented as an intermediate-risk gene, attributable to its critical role in the repair of DNA double strand breaks [39]. The *RAD50* variant (rs139372231) is a C to T base change at nucleotide position 1153 that results in an amino acid change from arginine to cysteine at codon 385. The RAD50 protein forms a complex with the meiotic recombination 11 (MRE11) and nibrin (NBN) double-strand break repair proteins, which bind to strands of damaged DNA and hold broken ends together during the repair process. This MRE11-RAD50-NBN complex is important to prevent accumulation of DNA damage that may trigger uncontrolled cellular division, in order to promote maintenance of genome stability [40]. Pathogenic mutations in the *RAD50* gene may lead to the production of an abnormally small, nonfunctional or missing RAD50 protein that may fail to respond effectively to DNA damage [41].

This study has demonstrated the feasibility of WES to achieve a high level of comprehensive genomic testing. The rare missense mutation Q85E, identified in the *MUC1* gene in the daughter of the index case with early-onset breast cancer, was absent in the mother and her affected cousin. MUC1 is a transmembrane heterodimeric oncoprotein that is highly expressed in various cancers and correlates with potential for malignancy [42]. Yamada et al. [41] reported that *MUC1* gene expression is regulated by DNA methylation and histone modification of the *MUC1* promoter. They postulated that an understanding of the epigenetic changes of *MUC1* may be of importance for prediction of risk and outcome for patients diagnosed with breast cancer. Deleterious germline mutations in the *MUC1* gene is a well-documented cause of medullary cystic kidney disease, also known as mucin-1 kidney disease [43]. In breast cancer, however, mutations in the *MUC1* gene may only present as somatic changes within tumour DNA, and are unlikely to be clinically useful in prediction of familial risk. Thus, detection of this germline *MUC1* mutation in the daughter may be considered an incidental finding in relation to kidney disease, if proven to be pathogenic in future studies.

A shift in our focus to low-penetrance gene variants highlighted the potential significance of folate pathway SNPs as a risk determinant for hormone receptor-positive breast cancer. Previous studies have demonstrated that the genotype distribution and allele frequencies of SNPs in the *MTHFR* gene differ significantly between population groups in South Africa [44]. The relatively high frequency of *MTHFR* 677 C>T is not only of concern for cancer risk, but also in development of other chronic diseases such as CVD. The SNP modifies the effect of hormone replacement therapy (HRT) on lipid and metabolic parameters in postmenopausal women [45]. Homozygosity (TT genotype) for *MTHFR* 677 C>T is associated with a statistically significant increase in both total and low-density lipoprotein (LDL) cholesterol levels, while presence of the C-allele is associated with a reduction in these lipid levels after one year of HRT. The favourable effect of HRT on lipid levels was reversed in *MTHFR* TT homozygotes.

The correlation of a given genomic risk profile with clinical and therapeutic outcomes as well as risk for drug toxicity is complicated by clinico-pathological heterogeneity as observed in the family studied here. The low-penetrance *MTHFR* 677 C>T mutation has previously been linked to the luminal subtype of breast cancer [15], in a similar way that *BRCA1* mutations predominate in patients with hormone receptor-negative breast cancer of the basal-type. *MTHFR* 677 C>T has also been implicated as a causative genetic determinant in altered expression of microRNAs (miRNAs) [46]. These short ≈22 nucleotide RNA regulators are involved in a wide spectrum of cellular processes involving epigenetic changes [47]. The overall importance of polymorphic variation remains high due to multiplicative effects, such that individuals possessing several functional polymorphisms may have a significantly increased risk of breast cancer [48,49,50]. The presence *of RAD50* R385C, in combination with *MTHFR* 677 C>T previously identified as a *BRCA*-modifier gene [51], is consistent with the modest family history reported by the index case.

Although functional SNPs do not confer significant individual risks for breast cancer, cumulative data supported by WES suggest that co-inheritance of multiple low-penetrance mutations underlying cancer pathways and rare variants in driver genes may explain the majority of non-*BRCA* familial disease [52,53]. Variation in folate-pathway genes such as *MTHFR*, *MTR* and *MTRR* may disrupt homeostasis and confer significant risk of breast cancer, attributable to impaired DNA repair [54,55]. Sangrajrang et al. [56] highlighted the role of genetic variation in the *MTR* and *MTRR* genes in relation to menopausal status of breast cancer patients. Co-inheritance of *MTHFR* 677C>T with high-penetrance causative mutations in the *BRCA1/2* genes may increase the risk of early-onset breast/ovarian cancer, while mutation 1298 A>C in the same gene is associated with sporadic breast cancer [51]. Elucidation of the role of *MTHFR* as a modifier gene in familial breast cancer is of particular relevance in South Africa, given the high frequency of founder *BRCA1/2* mutations in Afrikaner, Coloured and Xhosa breast cancer patients [29,30].

Current literature does not suggest a particular strategy with regards to surveillance and prophylactic management in patients with low-moderate risk variants [16]. However, combining pathway-based SNP genotyping with *BRCA* founder mutation screening where appropriate, may provide a viable risk reduction strategy for non-communicable diseases characterized by shared disease pathways [4,7]. Due to constantly evolving genetic knowledge, the ideal breast cancer susceptibility test remains elusive as it requires continuous development and refinement as new information on VUS classification and clinical relevance of functional polymorphisms become available in the scientific literature. The identification of actionable functional polymorphisms in important disease pathways covered to a certain extent in the CVD multi-gene assay, which was recently extended to include *CYP2D6* pharmacogenetics [28,31], caused a paradigm shift in our understanding of the pieces of the susceptibility puzzle. Implications for genetic counseling remains uncertain and can only be comprehended by incorporating fixed genetic and modifiable environmental risk factors into an existing body of knowledge. The PSGT strategy, which incorporates lifestyle factors with pathology and genetic test results, strives to facilitate patient selection and clinical interpretation [4]. Improved understanding of the genetic overlap between risk factors for ER-positive breast cancer and other chronic diseases of lifestyle warrants consideration of a polygenic approach to disease prevention.

Limitations of the study include the fact that WES evaluates only the subset of DNA regions that encode proteins (known as exons, or collectively the exome), which constitutes about 1% of the human genome. There are approximately 20,000 genes encoded by an estimated 180,000 exons, but due to the technological limitations of NGS previously reviewed [7], it is not feasible to completely cover every single gene with 100% certainty. Clinically relevant mutations could therefore be missed in cases where the gene region containing a disease-causing variant is not represented in the WES reads. Complex genetic insertions or deletions are also not reliably detected by WES. We have previously illustrated these problems in relation to the *CYP2D6* gene, which could be addressed by using a combination of different mutation detection methods to obtain reliable results [4]. A similar approach was followed in this study. Furthermore, it has been shown that every healthy person carries several deleterious mutations, without clinical manifestation of the associated medical condition due to gene–gene and gene–environment interaction [57]. For this reason, information on VUS confirmed by Sanger sequencing is stored in a database to gain knowledge from a larger number of samples in future.

The strength of this study lies in capturing genetic, pathology and lifestyle information before and after WES to formulate pathway-based algorithms for breast cancer treatment and prevention. The important role of gene–gene and gene–environment interaction has been emphasised previously to explain the missing heritability in breast cancer. However, to our knowledge, this is the first study to demonstrate the importance of functional SNPs in the *MTHFR* gene that could be further explored by WES to confirm or support clinical relevance.

## 4. Materials and Methods

### 4.1. Ethics Approval

In response to a needs assessment survey amongst healthcare professionals in South Africa, the PSGT platform was established to combine service delivery with health outcome studies [58]. Approval for this study and use of the genomics database was obtained from the Human Research Ethics Committee of Stellenbosch University (reference number N09/08/224).

### 4.2. Study Population

Three members of the pedigree of the index case were included in this study. The index patient diagnosed with breast cancer at the age of 57 years was referred for the MammaPrint test performed in 2008. She forms part of the cases included in the study of Grant et al. [10], who made special mention of this patient due to a low-risk MammaPrint result for both tumours (invasive ductal and lobular carcinoma). Her daughter was diagnosed with invasive ductal carcinoma approximately one year later, at the age of 29 years. A cousin was diagnosed with invasive lobular carcinoma at the age of 42 years. The index case experienced severe drug side-effects with use of tamoxifen, including vaginal atrophy and hemorrhagic cystitis. She also reported folate supplementation to alleviate side-effects during methotrexate treatment for arthritis.

A total of 164 unrelated female breast cancer patients (Table 2) and 160 controls without a family history of breast cancer were included for extended mutation screening of variants detected by WES. Except for a high BMI, the index case did not report any of the lifestyle risk factors listed in Table 2. Periodic high alcohol consumption was reported in the affected daughter with early-onset breast cancer treated with both tamoxifen and chemotherapy.

Patients with benign neoplasms of the breast or other malignancies were excluded from the study. Only unrelated breast cancer patients with known ER status who signed informed consent for genetic studies were selected for high-throughput genotyping, comprising 60 (37%) Coloured patients of mixed ancestry and 104 (63%) Caucasians, including the index case. Patients with histopathology confirmed breast cancer were recruited at the Tygerberg Hospital Breast Cancer Clinic or referred from private practicing clinicians, as previously described by van der Merwe et al. [28]. Patients referred by clinicians in private practice participated in a chronic disease screen using the CVD multi-gene assay [59] as the core genetic component, recently extended to include *CYP2D6* pharmacogenetics [31]. An online questionnaire was used to obtain information on personal and family medical conditions, pathology, medication use/side effects and lifestyle factors to enable clinical interpretation of the genetic findings. Both the index patient and her affected 29-year old daughter tested positive for *MTHFR* 677 C>T, investigated in this study as a potential contributing factor for breast cancer development against a possible genetic background of rare variants that may be uncovered by WES. In this family with inherited breast cancer, both the daughter (BMI 27.5 kg/m^2^) and her mother (BMI 27.8 kg/m^2^) were overweight with suboptimal intake of folate-rich food in the diet (folate score < 14) according to the nutrition and lifestyle assessment performed as previously described by Delport et al. [27]. The presence of *BRCA1* and *BRCA2* mutations was largely excluded as the cause of familial breast cancer using NGS and multiplex ligation-dependent probe amplification (MLPA) at a commercial laboratory (NewGene, Newcastle, UK).

### 4.3. DNA Extraction

DNA was extracted from whole blood using the spin protocol of QIAGEN QIAamp^®^ DNA Blood Mini Kit (Hilden, Germany) or Oragene reagents (Ottawa, ON, Canada) for saliva specimens, according to the instructions provided with these commercially available kits. The NanoDrop^®^ ND-1000 Spectrophotometer (Nanodrop Technologies, Wilmington, NC, USA) and its v3.5.2 software package was used to measure the DNA quality and quantity. All genomic DNA samples were diluted to a final concentration of 10 ng/µL using nuclease-free water.

### 4.4. Whole Exome Sequencing

**Figure 5:**
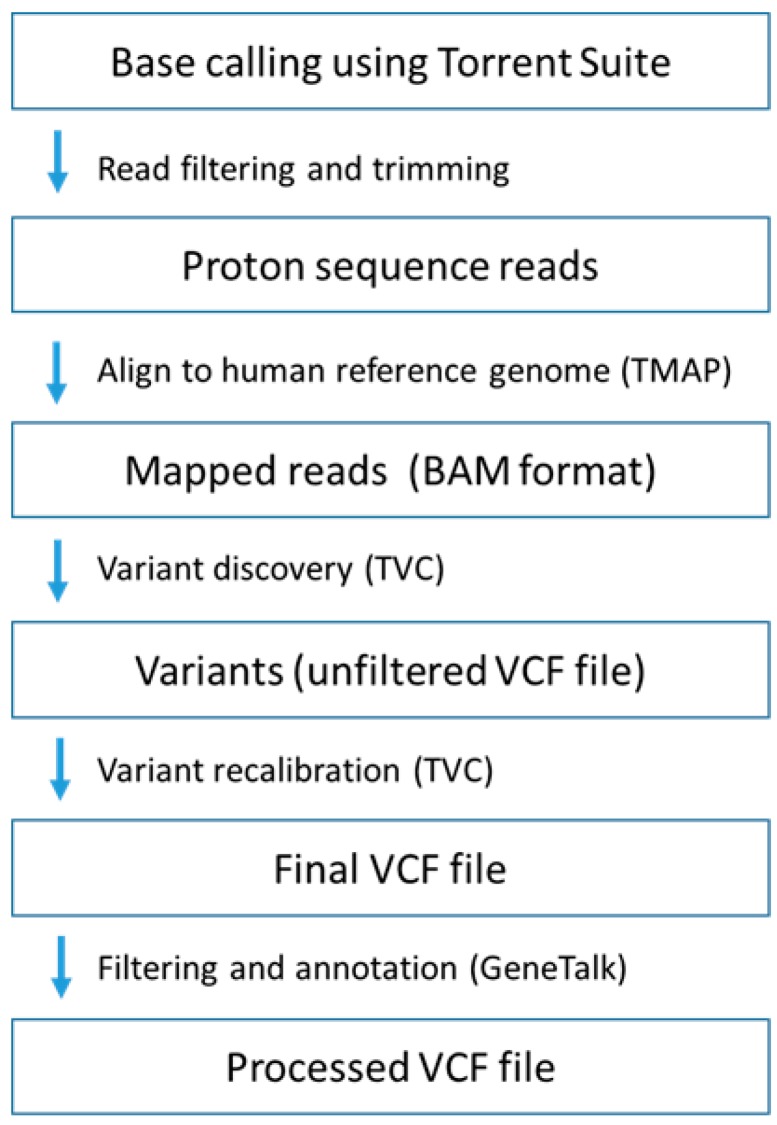
A simplified representation of the data analysis pipeline used by Torrent Suite for the identification of variants in WES data. Base calling, read filtering and trimming were performed by the Torrent Suite software. The torrent mapping alignment (TMAP) [60] program was used to map the resulting reads to the human reference genome. Variant calling was performed by the Torrent Variant Caller (TVC) in three steps: generate candidates, evaluate candidates and filter candidates. Final filtering and annotation of variant call files (VCF) were performed using GeneTalk [62] web-based bioinformatics tool.

WES was performed at the Central Analytical Facility (Stellenbosch University, Stellenbosch, South Africa) using the Ion Proton™ System (Thermo Fisher Scientific, Waltham, MA, USA) for NGS. The Ion AmpliSeq™ Exome RDY Library Preparation protocol was used (Publication Number MAN0010084) and template amplification was performed using the Ion PI™ Template OT2 200 Kit v3 (Thermo Fisher Scientific, Waltham, MA, USA) (Publication Number MAN0009133). Semi-conductor sequencing on the Ion Proton system was performed using the Ion PI™ Sequencing 200 Kit v3 (Publication Number MAN0009136) with the Ion PI™ Chip Kit v2. This method is designed to target all human exons. The sequencing run time on the Ion Proton™ System was approximately 4 h which enabled the entire workflow from library construction to primary data analysis and result generation to be done in under 24 h. The Torrent Suite™ (Thermo Fisher Scientific, Waltham, MA, USA) proprietary software is the standard program through which this NGS apparatus operates and offered simple run setup using predesigned workflows and run monitoring in real-time. WES analysis comprised several intermediate steps involved in the read-processing pipeline, as illustrated in Figure 5.

First, sequence reads were aligned to the human reference genome (hg19) using the torrent mapping alignment (TMAP) program [60]. This was followed by read analysis and processing using the preinstalled Torrent Suite™ Software and variant calling with the Torrent Variant Caller (TVC) plug-in. Mapped reads were processed in binary alignment/map (BAM) and indexed sequence alignment/map (SAM) file format. TVC was used for functional annotation and filtering of variant output in variant call files (VCFs). The finding that hg19 contains minor alleles at >1.5 million loci led to repeat mapping of the reads to a synthetic Caucasian major allele reference sequence (CEU-MARS) [61], following the same process illustrated in Figure 5. Resulting VCFs were then processed using GeneTalk [62], a web-based tool for filtering and annotation of uploadable VCFs.

A coverage depth of 15× was used for detection of potentially causative gene variants, prior to confirmation by Sanger sequencing. Coverage provides counts of read depth (DP) at two different levels; the sample level where the DP value is the count of reads that passed the caller’s internal quality control metrics and at the site level where this value is the unfiltered depth over all samples. In addition to the aforementioned quality filters applied, rare variants were filtered on a population frequency of <0.01% with variant function set to exclude all synonymous variants. A list containing 409 genes from the Ion AmpliSeq™ Comprehensive Cancer Panel (Thermo Fisher Scientific, Waltham, MA, USA; catalog number 4477685) was used to search for high impact mutations as well as polymorphic variation in folate-related genes included in the panel. Bioinformatics tools freely available on the internet (Table 1) were used to predict the functional effects for variants of interest.

### 4.5. Sanger Sequencing and Real-Time Polymerase Chain Reaction (PCR)

Following the identification of potentially causative variants or functional SNPs involved in the folate pathway with use of WES, confirmation was performed by Sanger sequencing. Selected variants were screened for in controls (*n* = 160) and the total breast cancer sample (*n* = 164) using real-time PCR. Two microliters of template DNA (10 ng/µL) was used in a total reaction volume of 10 µL. Applied Biosystems^®^ (ABI™) (Foster City, CA, USA) *Taq*Man^®^ SNP Genotyping Assays were used for the real-time PCR runs according to standard *Taq*Man^®^ SNP Genotyping protocols. Pre-designed 40× assay mix consisted of unlabelled primers and *Taq*Man^®^ Minor Groove Binder (MGB) probes (VIC**^®^** and FAM™ dye-labelled). These assays were used for end-point genotyping by allelic discrimination analysis for SNPs on the Corbett Rotor-Gene™ 6000 (Mortlake, New South Wales, Australia) or Roche LightCycler^®^ 480 II (Basel, Switzerland). The oligonucleotide primer sequences used for Sanger sequencing and the *Taq*Man assays employed in this study are presented in Table 3.

## 5. Conclusions

This study describes a framework for WES performed alongside clinical and pathology assessments to provide a clear strategy for the way forward. We moved from single-gene analysis of *BRCA1/2* founder mutations in the high-risk South African population to multi-gene panel testing, meant to assist clinicians in the decision to pursue WES. The selection of the index patient was primarily based on the need to establish the role of *MTHFR* 677 C>T in familial risk, due to the potential for cancer prevention at the population level. Our results support previous findings indicating that the majority of genetically uncharacterised breast cancer cases may be caused by a combination of low–moderate penetrance mutations exerting their effect against a high-risk clinical background. To our knowledge, this is the first study using WES to investigate the significance of folate pathway SNPs as risk reduction targets beyond *BRCA1/2* in familial risk. WES preceded by PSGT facilitated the identification and clinical interpretation of genetic risk factors of relevance to both cancer development and tailored therapeutic intervention in a single test.

## Figures and Tables

**Table 1:** Bioinformatics analysis of missense mutation *RAD50* R385C as a variant of uncertain clinical significance.

Bioinformatics Tool Applied	Predicted Biological Effect
Sorting Intolerant From Tolerant (SIFT)	Damaging (rank score = 0.7209)
Polyphen	Benign (score = 0.339)
Variant Effect Predictor	Moderate impact
MutationTaster	Disease causing (rank score = 0.8103)
MetalR	Tolerated (rank score = 0.2311)
Provean	Neutral (rank score = 0.4681)
Likelihood Ratio Test	Deleterious (rank score = 0.5373)

**Table 2:** Characteristics of 164 female breast cancer patients subdivided according to tumour pathology into estrogen receptor (ER) positive (*n* = 49) and negative (*n* = 115) cases.

Variables		ER-Negative	ER-Positive
Median (Range)	Number	49	115 *
Age (median, range)	164	49 (30–77)	54 (31–83)
Body mass index	146	25 (17–47)	26 (17–41)
**Count (Percentage)**			
Ethnicity ^†^	164	22 (45)	38 (33)
Family history of cancer	163	30 (61)	59 (52)
Hormone replacement therapy	163	9 (19)	15 (13)
Oral contraceptives	163	20 (42)	32 (28)
Current smoking	160	17 (35)	34 (30)
High alcohol consumption	156	25 (52)	62 (57)

* Includes the index case; ^†^ Percentage of non-Caucasian patients are indicated in brackets.

**Table 3:** Oligonucleotide primers used for conventional polymerase chain reaction (PCR) and Sanger sequencing, and the identification numbers of commercially available *Taq*Man assays used for real-time PCR.

Gene	dbSNP ID#	Primer	Oligonucleotide Primers for Sanger Sequencing	Size (bp)	*Taq*Man Assay ID Numbers
*MTHFR*	rs1801133	Forward	ATCCCTCGCCTTGAACA	256	C_1202889_20
		Reverse	TCACCTGGATGGGAAAGAT		
*MTR*	rs1805087	Forward	GAACATCCCAAGCCCAC	595	C_12005959_10
		Reverse	CACCTGTTTCCCTGCTG		
*MTRR*	rs1801394	Forward	GTTTCATTCGTACACTCTCC	616	C_3068176_10
		Reverse	CAGCATATGCTACTTCTGTC		
*RAD50*	rs139372231	Forward	ATCCACATGCTCAGGGGTAC	528	C_171053490_10
		Reverse	GCCAAAATGGAGTCCAACC		
*MUC1*	rs773704188	Forward	ATTCCCAGCCACCACTCTGA	493	Not available
		Reverse	CCCAACCTTAAGTGCACCAGT		

bp: base pair, dnSNP: database of single nucleotide polymorphisms.

## Abbreviations

ABI	Applied Biosystems
BAM	Binary alignment/map
BMI	Body mass index
CEU-MARS	Caucasian major allele reference sequence
CVD	Cardiovascular disease
CYP	Cytochrome P450
dnSNP	Database of Single Nucleotide Polymorphisms
ER	Estrogen receptor
hg	Human reference genome
HRT	Hormone replacement therapy
IGV	Integrative Genome Viewer
MINDACT	Microarray In Node negative Disease may Avoid Chemo Therapy
MRC	Medical Research Council
MRE11	Meiotic recombination 11
MTHFR	Methylene tetrahydrofolate reductase
MTR	Methionine synthase
MTRR	Methionine synthase reductase
MUC1	Mucin 1
NBN	Nibrin
NGS	Next generation sequencing
NRF	National Research Foundation
PCR	Polymerase chain reaction
PR	Progesterone receptor
PSGT	Pathology-supported genetic testing
RAD50	Double strand break repair protein
SAM	Sequence alignment/map
SHIP	Strategic Health Innovation Partnerships
SNPs	Single nucleotide polymorphisms
TMAP	Torrent mapping alignment
TVC	Torrent Variant Caller
VCF	Variant call files
VUS	Variant of uncertain clinical significance
WES	Whole exome sequencing
